# Efficiency and equity of bed utilization in China’s health institutions: based on the rank-sum ratio method

**DOI:** 10.1186/s12939-023-01986-4

**Published:** 2023-09-02

**Authors:** Hang Zhang, Leiyu Shi, Junyan Yang, Gang Sun

**Affiliations:** 1 Department of Health Management, School of Health Management, Southern Medical University, Guangzhou, 510515 Guangdong China; 2 Department of Health Policy and Management, Bloomberg School of Public Health, Johns Hopkins University, Baltimore, MD 21205 USA

**Keywords:** Efficiency and equity of bed utilization, China's health institutions, Rank-sum ratio method

## Abstract

**Objective:**

The study aimed to analyze the efficiency and equity of bed utilization in Please check if the section headings are assigned to appropriate levels.China’s healthcare institutions and to compare and analyze the overall health resource utilization efficiency in recent years and some specific utilization conditions in 2021, to provide empirical experience for the allocation of health care resources in epidemic China.

**Methods:**

To compare and analyze the overall health resource utilization efficiency of the whole country with that of the East, middle, and West in 2021, and to analyze the bed utilization efficiency of different types of healthcare institutions in China and the bed utilization efficiency of various types of specialist hospitals in the country in 2021 by using the rank-sum ratio method.

**Results:**

In 2021, the bed utilization rate of China’s health institutions was 69.82%, and the number of bed turnover times was 27.65 times; the bed utilization rate of hospitals was 74.6%, and the number of bed turnover times was 26.08 times. The number of hospital bed turnovers was highest in the western region, lowest in the central region, and close to the national average in the eastern region. The average length of stay for discharged patients was the highest in the central region, the lowest in the eastern region, and the same as the national average in the western region. The analysis of rank-sum ratio method shows that among different types of health institutions’ bed utilization efficiency (*r* = 0.935, *P* = 0.000), general hospitals and traditional Chinese medicine hospitals have the best bed utilization rate, and the bed utilization rate of community health service centers (stations) needs to be improved; while among various types of specialized hospitals’ bed utilization efficiency (*r* = 0.959, *P* = 0.000), oncology hospitals, thoracic hospitals, and hematology hospitals, children’s hospitals have high bed utilization efficiency; leprosy hospitals, cosmetic hospitals, and stomatology hospitals have low bed utilization efficiency. Health technicians per 1,000 population are highest in the western region, lowest in the central region, and lower in the eastern region than in the western region but slightly higher than the national average. The number of beds in health institutions per 1,000 population is the highest in the central region, the lowest in the eastern region, and slightly lower in the northwest than in the central region but higher than the national average.

**Conclusion:**

China’s investment in health funding in the field of health care has been on the rise in recent years. However, there still exists the situation of uneven investment in health expenses and inconsistent medical efficiency among regions. And change such a status quo can be further improved in terms of government, capital, human resources, technology, information system, and so on.

## Introduction

 With the rapid development of the economy and society, people’s demand for health is also increasing. In 2022, the General Office of the State Council issued the “14th Five-Year Plan” for national health, which identified the “health priority, sharing; prevention first, strengthening the grassroots; improve quality, promote balance; reform and innovation, System Integration” and puts forward the basic principle of “putting the safety of people’s lives and physical health in the first place”. To meet the nation’s growing health needs, it is particularly important to rationalize the use of limited health resources and enhance the efficiency and fairness of their utilization. There is still a big gap between the development of medical and health care in China and the people’s health needs and the requirements of economic and social development, and there is still the problem of imbalance and insufficiency, and there is still the problem of insufficient total amount of resources in health care institutions, irrational configuration structure, and inefficient utilization of resources. How to utilize healthcare resources more efficiently, how to improve the service quality and efficiency of healthcare institutions, and how to give full play to the decisive role of the market in the allocation of resources will be important elements in the better implementation of the goals set out in the 14th Five-Year National Health Plan. The bed utilization rate is an important indicator for judging the utilization of healthcare resources. An analysis of the bed utilization rate of each healthcare institution will help to evaluate the efficiency of healthcare institutions and the allocation of resources. Based on this analysis, relevant recommendations and countermeasures are put forward, which help to improve the efficiency of the utilization of medical and health resources and the fairness of their allocation, and thus provide people with more high-quality, efficient, convenient, and adequate medical and health services.

## Materials and methods

### Data collection

In this paper, the total number of actual open bed days (days), the average number of open beds (beds), the total number of bed days occupied by discharged patients (days), the average hospitalization days of discharged patients (days), the utilization rate of beds in various types of health institutions (%), the average number of working days for beds (days), the number of turnover times of beds (times), the number of health technicians per 1,000 population (persons), the number of beds per 1,000 health institutions (beds), Per capita total health expenditure (yuan), average number of consultations per doctor per day (person-times), and average number of inpatient bed-days per doctor per day (days), etc., were obtained from the National Bureau of Statistics and the 2022 China Healthcare Statistics Yearbook [1]. The relevant definitions and indicators of bed utilization efficiency and equity in healthcare institutions mentioned therein are as follows: (1). The total number of bed days opened in healthcare institutions: the sum of the number of beds opened at midnight each night of the year in each department of the hospital, regardless of whether or not the bed is occupied by a patient, should be included in the calculation. Including beds suspended for sterilization and minor repairs, etc., and extra beds for more than six months. It does not include the beds that are out of service due to ward expansion or major repairs, or the temporary addition of beds (2). Average number of open beds: i.e., the total number of bed days opened/the number of calendar days in the current year, which is used as one of the data to measure the number of turnover times of beds; (3). Total number of bed days occupied by discharged persons: refers to the total number of inpatient bed days for all discharged persons. This includes the number of inpatient bed days for normal deliveries, undischarged discharges, inpatient discharges after checkups, untreated discharges, and normal discharges of healthy people after abortions or sterilizations (4). Average hospitalization days of discharged patients: i.e. the total number of bed days occupied by discharged patients / the number of discharged patients. It is a hard and comprehensive indicator for evaluating medical efficiency and effectiveness, medical quality, and technical level (5). Bed utilization rate: i.e. the total number of bed days occupied/the total number of bed days opened*100% (6). Bed working days: i.e. total bed days occupied/average number of open beds. It is generally used to measure the utilization of hospital beds, and also belongs to one of the indicators reflecting the quality of the hospital’s work; (7) the number of turnover of beds: i.e., the number of discharges/average number of open beds (8) health professionals per 1,000 population: that is, the number of health professionals/population * 1000; (9) the number of beds per 1,000 population in health care institutions: that is, the number of beds in health care institutions/population * 1000 (10) per capita total cost of health: that is, the total cost of health in each region/population in each region (11). Average total cost of health per bed: i.e. total cost of health in each region/number of beds in health institutions in each region (12). Average number of consultations per doctor per day: i.e., number of consultations/average number of doctors/251 (13). Average number of inpatient bed days per doctor per day: i.e., total number of bed days occupied/average number of doctors/365 (14). Population number is the resident population of the National Bureau of Statistics.

### Research methods

The rank sum test is mainly used for comparative testing of paired, grouped, diverse or hierarchical information. Its main advantages are that it is not restricted by the overall distribution and is widely applicable; it applies to rank information and information with no definite value at both ends; and it is easy to understand and easy to calculate. In this study, by collecting information related to bed utilization in healthcare institutions in China in 2021, the efficiency and fairness of bed utilization in all aspects of healthcare institutions were categorized and comprehensively evaluated, so the rank-sum ratio method of the rank-sum test was chosen.

Rank-sum ratio (RSR method short) is a statistical analysis method proposed by Prof. Tian Fengtiao, a Chinese scholar and former professor of the Chinese Academy of Preventive Medical Sciences, in 1988, which combines the respective advantages of classical parametric statistics and modern non-parametric statistics, and it is not only applicable to the comprehensive evaluation of four-compartment table data, but also applicable to the comprehensive evaluation of row x list data, and at the same time applicable to the comprehensive evaluation of measurement data and categorized data. It is not only applicable to the comprehensive evaluation of four-cell table information but also applicable to the comprehensive evaluation of row x list information, as well as applicable to the comprehensive evaluation of measurement and classification information [2, 3]. It ranks benefit-oriented indicators from small to large and cost-oriented indicators from large to small, then calculates rankings and ratios, and finally performs statistical regression and ranking. The dimensionless statistic RSR is obtained through rank conversion, and the evaluation object is directly ranked or sorted according to the RSR value, to provide a comprehensive evaluation of the evaluation object.

The main research indicators used in this study include average workdays of beds, number of bed turnovers, bed utilization rate, and average length of stay of discharged patients. Among them, the average working days of beds, the number of bed turnovers, and the bed utilization rate are high superiority indicators, which need to be sorted from small to large. The average length of stay of discharged patients is a low-optimal indicator and needs to be ranked from largest to smallest [4, 5]. The formula for calculating the rank-sum ratio is as follows:

$$RSR=\sum\limits_{t=1}^BRij/\left(B\ast A\right)$$

### Statistical analysis

In this paper, the data were processed by Excel2019, and the linear regression analysis was carried out by SPSS software.

## Results

### National health resource investment, 2017–2021

**Fig. 1:**
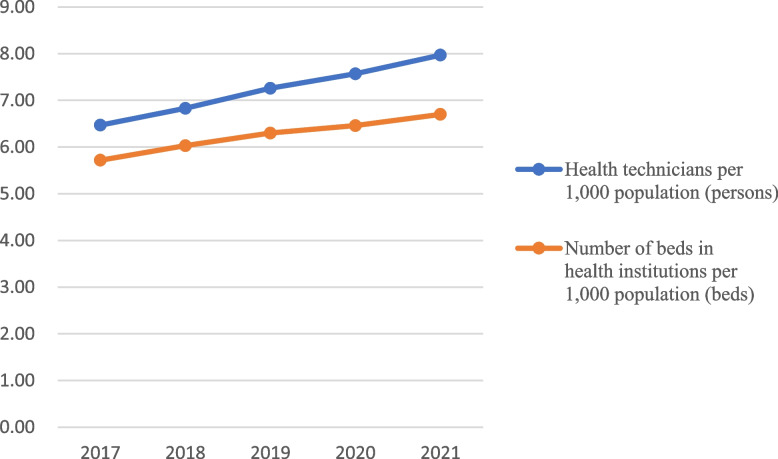
National comparison of health technicians and beds per 1,000 population, 2017–2021

**Fig. 2:**
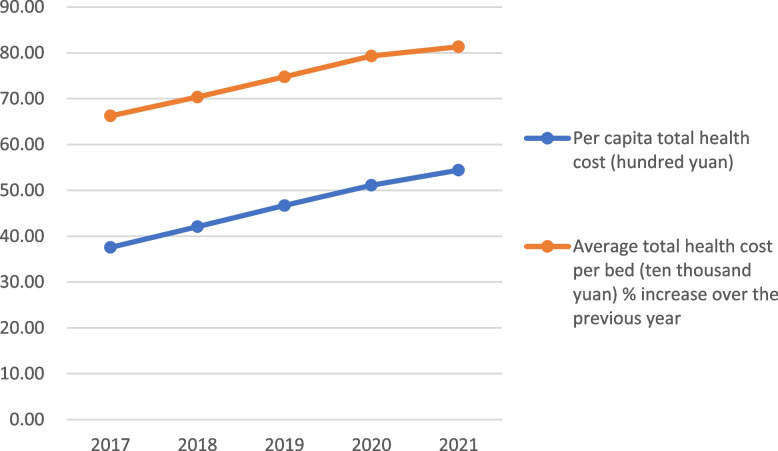
Comparison of national total health costs per capita and per bed, 2017–2021

The data in Table 1 shows that in recent years, China has been increasing its investment in the development of health care, both in terms of the training of health professionals, the increase in the number of beds in health care institutions, and the financial investment in health care, year by year. Since 2017, health technicians per 1,000 population have been increasing at a rate of 5.43% per year, and the number of beds in healthcare institutions has increased at a rate of 4.53% per year. The total health cost per capita investment is growing at an average annual growth rate of 10.34%. See Figs. 1 and 2.

**Table 1:** National health technicians, health care facility beds, and health cost inputs, 2017–2021

Year	Health technicians per 1,000 population (persons)		Number of beds in health institutions per 1,000 population (beds)		Per capita total health cost (hundred yuan)		Average total health cost per bed (ten thousand yuan) % increase over the previous year	
		Increase over the previous year		Increase over the previous year		Increase over the previous year		Increase over the previous year
2017	6.47	5.72	5.72	6.52	37.57	12.86	66.24	5.92
2018	6.83	5.56	6.03	5.42	42.07	11.98	70.35	6.20
2019	7.26	6.30	6.30	4.48	46.69	10.98	74.76	6.27
2020	7.57	4.27	6.46	2.54	51.11	9.47	79.31	6.08
2021	7.97	5.28	6.70	3.72	54.40	6.44	81.32	2.53
Average % increase per year		5.43		4.53		10.34		5.40

### Status of national health resources utilization efficiency in 2021

The data shows that in 2021, the total number of visits to health institutions nationwide amounted to 8.472 billion, a year-on-year increase of 9.45%, of which 8.041 billion were outpatient emergencies and 247 million were hospitalizations. The total number of visits to all types of hospitals nationwide was 3.884 billion, an increase of 16.8% over the previous year, of which 3.782 billion were outpatient emergencies. The number of hospitalized patients in medical institutions nationwide was 201.551 million, an increase of 18.031 million over 2020. The average daily number of consultations by physicians in regional general hospitals was 7.2, and the average daily number of inpatient bed days was 2.1.

The total number of bed days actually opened in healthcare institutions nationwide was 3,252,810,000, with an average of 8,912,000 open beds; the total number of bed days occupied was 2,253,441,000, of which 2,158,696,000 were occupied by discharged patients; the number of bed turnovers was 27.65, with a bed workday of 2,520,000 days, and the bed occupancy rate of 69.82%, with an average number of hospitalization days of 8.8 days. The number of bed turnovers in hospitals in all regions of the country was 26.08, the number of bed working days was 272.3, the bed utilization rate was 74.6%, and the average number of days of hospitalization was 9.2 days. The data shows that the bed working days and bed utilization rate of hospitals in all regions are higher than the average of healthcare institutions See Table 2.

**Table 2:** Hospital bed utilization by region, 2021

Region	Number of turnover of beds(times)	Bed utilization rate (%)	Average bed working days (days)	Average hospitalization days of discharged patients (days)
Nationwide	26.08	74.6	272.3	9.2
East	25.86	74.2	270.8	9.1
Central part	24.91	73.6	268.7	9.5
West	27.65	76.2	278.3	9.2

### Analysis of the utilization efficiency of hospital beds nationwide and in the eastern, central and western regions in 2021

**Fig. 3:**
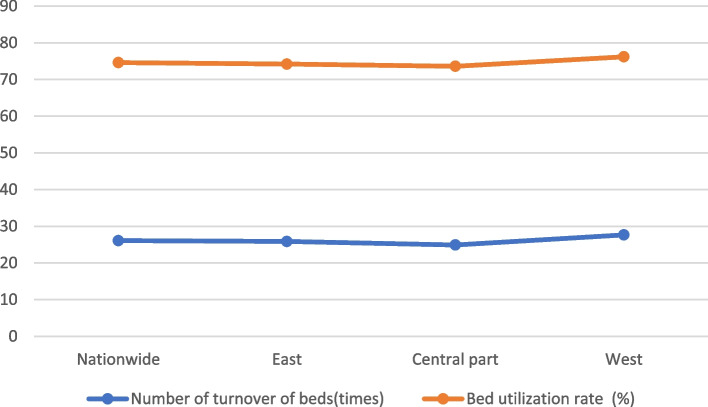
Comparison of the number of bed turnovers and utilization rates by region, 2021

The data in Table 2 show that: from the viewpoint of the number of turnover of hospital beds, the highest in the western region is 27.65 times, the lowest in the central region is 24.91 times, and the eastern region is 25.86, close to the national average of 26.08; from the viewpoint of the utilization rate of hospital beds, the highest in the western region is 76.2%, the lowest in the central region is 73.6%, and the eastern region is close to the national level of 74.2%; from the viewpoint of the average number of days of hospitalization for the discharged In terms of the average number of days of hospitalization for discharged patients, the highest in the central region is 9.5 days, the lowest in the east is 9.1 days, and the western region is on par with the national level at 9.2 days. The western region has a high utilization rate of hospital bed turnover, but the average length of stay of discharged patients is at a medium level, which may be related to the living hygiene habits of people in the western region. The number of turnover of hospital beds and the utilization rate of hospital beds are the same in all regions See Fig. 3.

### Analysis of medical and health resources input by region in 2021

**Fig. 4:**
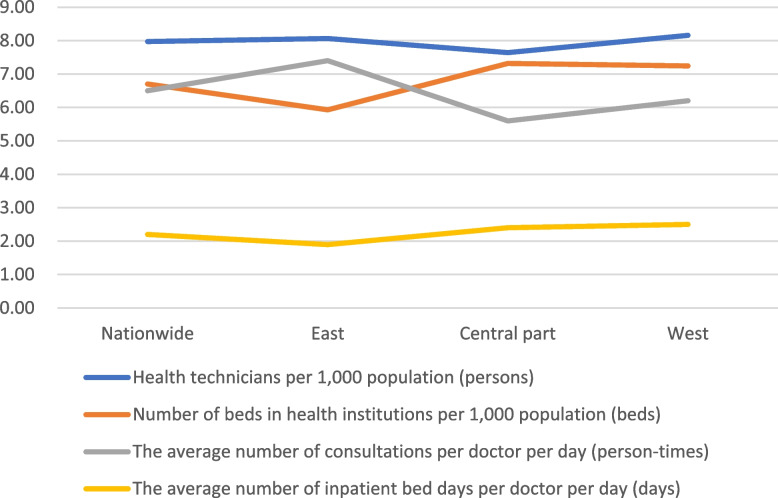
Comparison of health technicians and beds per 1,000 population and the average number of consultations and bed days carried out by physicians per day, nationally and in the eastern, central, and western regions, 2021

Table 3 shows that: from the point of view of the number of health technicians per 1,000 population, the number of people in the eastern and western regions is close to the number of 8.06 and 8.16, respectively; in the central region, the number is relatively small at 7.64. From the point of view of the number of beds in health institutions per 1,000 population, 5.93 beds in the eastern region are the least; the central and western regions are close to each other, respectively 7.32 and 7.24 beds; from the point of view of the number of doctors’ daily average number of consultations and treatments, the highest in the eastern region is 7.4, the lowest in central China is 5.6, and in the western region it’s 6.2, which is close to the national average level of 6.5. From the point of view of the number of hospitalization beds that are taken care of by the doctor, the average number of days is 7.4, which is close to the national average. From the point of view of the average daily number of inpatient beds taken care of by physicians, the highest in the western region is 2.5 days, the lowest in the eastern region is 1.9 days, and the lowest in the central region is 2.4 days, which is close to the national average level of 2.2 days. The above indicators do not fluctuate much in the eastern, central, and western regions (see Fig. 4), indicating that the investment of medical and health resources in each region is balanced. At the same time, physicians in the East have a higher average number of consultations per day, but a lower average number of inpatient bed days per day, indicating that medical care in the East is highly efficient.

**Table 3:** Statistics on health care resources by region, 2021

Region	Population (10,000)	Health technicians per 1,000 population (persons)	Number of beds in health institutions per 1,000 population (beds)	The average number of consultations per doctor per day (person-times)	The average number of inpatient bed days per doctor per day (days)
Nationwide	141,260	7.97	6.70	6.5	2.2
East	60,834	8.06	5.93	7.4	1.9
Central part	41,945	7.64	7.32	5.6	2.4
West	38,281	8.16	7.24	6.2	2.5

### Analysis of bed utilization efficiency of different types of healthcare institutions nationwide in 2021

**Fig. 5:**
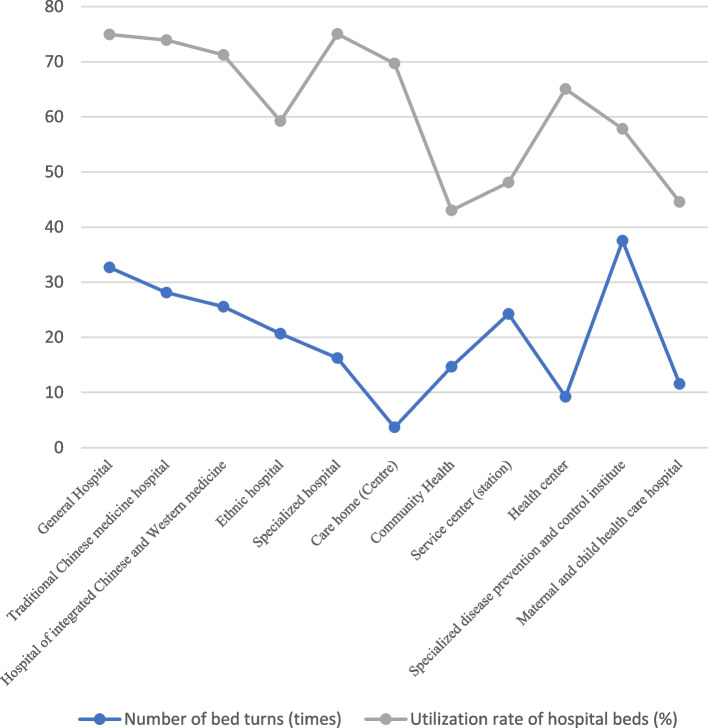
Comparison of the number of bed turnovers and utilization rates of different types of healthcare facilities nationwide, 2021

In terms of the number of bed turnovers in different types of health institutions, the average number of turnovers in nursing homes (centers) of 3.68 times, and the average number of turnovers in specialized disease prevention and treatment hospitals (institutes and stations) of 9.22 times are the least, i.e., the slowest turnovers, while the average number of turnovers in women’s and children’s health hospitals (institutes and stations) of 37.54 times and the average number of turnovers in general hospitals of 32.69 times are the most, which is the fastest turnovers. In terms of the two indicators of average bed working days and bed utilization rate, specialty hospitals (273.9, 75.05%) and general hospitals (273.5,74.94%) have the largest values, indicating high utilization efficiency; community health service centers (stations) (157.1,43.05%) and nursing homes (162.6,44.55%) have lower values, indicating a bottom utilization rate. In terms of the average length of stay of patients, maternal and child health hospitals (outpatient clinics and medical stations) had the shortest average of 5.4 days, nursing homes (centers) had the longest average of 52.8 days and specialized disease prevention and treatment hospitals (institutes and stations) had the longest average of 23.4 days See Table 4; Fig. 5.

**Table 4:** Utilization of hospital beds in different types of healthcare institutions nationwide, 2021

Mechanism classification	Number of bed turns (times)	Rank	Average hospital bed working days (days)	Rank	Utilization rate of hospital beds (%)	Rank	The average length of stay (day)	Rank	Rank Sum Ratio	Probit
General Hospital	32.69	10	273.5	10	74.94	10	8.2	9	0.9580	7.000
Traditional Chinese medicine hospital	28.12	9	269.8	9	73.92	9	9.3	8	0.9060	6.335
Hospital of integrated Chinese and Western medicine	25.55	8	260.0	8	71.24	8	9.9	6	0.8397	5.908
Ethnic hospital	20.67	6	216.2	5	59.23	5	9.9	5	0.5942	5.114
Specialized hospital	16.25	5	273.9	11	75.05	11	15.7	3	0.8295	5.605
Care home (Centre)	3.68	1	254.4	7	69.69	7	52.8	1	0.5616	4.651
Community Health Service center (station)	14.68	4	157.1	1	43.05	1	9.8	7	0.2673	3.665
Health center	24.25	7	175.6	3	48.10	3	6.6	10	0.4299	4.395
Specialized disease prevention and control institute	9.22	2	237.5	6	65.06	6	23.40	2	0.5868	4.886
Maternal and child health care hospital	37.54	11	211.0	4	57.82	4	5.40	11	0.6957	5.349
Sanatorium	11.56	3	162.6	2	44.55	2	11.10	4	0.2694	4.092

RSR was found to be significantly positively and linearly correlated with Y (*r* = 0.935, *P* = 0.000) and the linear regression equation was: RSR=-0.587 + 0.235Y by using instrumental analysis.

It was found that RSR had a significant positive linear correlation (*r* = 0.935, *P* = 0.000) with Y, and the linear regression equation was: RSR=-0.587 + 0.235Y. RSR was used to categorize the utilization efficiency of beds in different types of healthcare institutions in China in 2021. The results show that the bed utilization efficiency rank-sum ratios of general hospitals and Chinese medicine hospitals are ≥ 0.82, which are high utilization efficiency; the bed utilization efficiency rank-sum ratios of combined Chinese and Western medicine hospitals, ethnic hospitals, specialized hospitals, nursing homes (centers), health centers, specialized disease prevention and treatment hospitals (stations), maternity and child health care hospitals (stations), and sanatoriums are between 0.35 and 0.82, which are general utilization efficiency; the rank-sum ratios of community health service centers (stations) and community health centers (stations) are between 0.35 and 0.82, which are average utilization efficiency; and the bed utilization efficiency of community health centers (stations) is between 0.35 and 0.82, which is general utilization efficiency. The rank-sum ratio of bed utilization efficiency of service centers (stations) is ≤ 0.3531, which belongs to low utilization efficiency See Table 5.

**Table 5:** Classification of bed utilization efficiency of different types of health institutions nationwide, 2021

Category	Rank Sum Ratio(RSR)	Y(Probit)	Specific classification
Category I (high utilization efficiency)	≥ 0.82	≥ 6	General Hospital, Traditional Chinese Medicine hospital
Category II (general utilization efficiency)	> 0.35 and <0.82	> and < 6	Hospital of integrated Chinese and Western medicine, Ethnic hospital, Specialized hospital, Care home (Centre), Health center, Specialized disease prevention and control institute, Maternal and child health care hospitals (stations), Sanatorium
Category III (Low utilization efficiency)	≤ 0.3531	≤ 4	Community Health Service Center (station)

### Classification analysis of bed utilization efficiency of various types of specialized hospitals in 2021

**Fig. 6:**
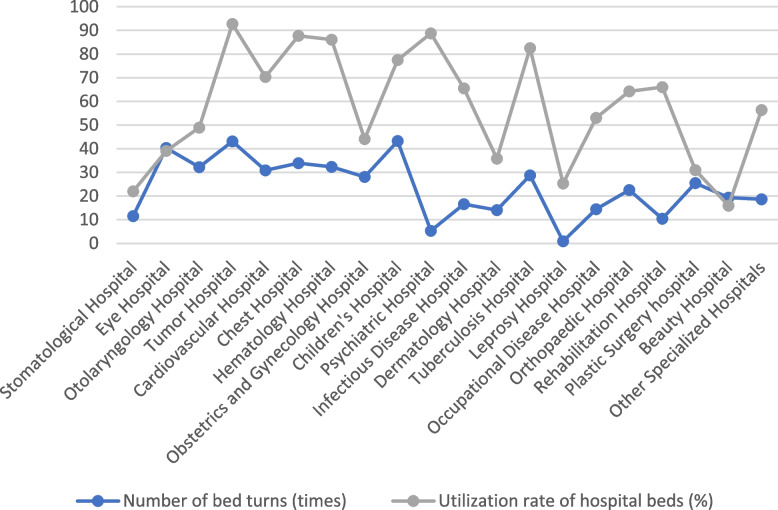
Comparison of bed turnover times and utilization rates of various types of specialized hospitals nationwide in 2021

The analysis results show that in terms of the number of bed turnover times, children’s hospitals average 43.23 times, oncology hospitals average 43.08 times, and ophthalmology hospitals average 40.31 times, which have faster bed turnover; leprosy hospitals average 0.88 times, psychiatric hospitals average 5.29 times, and rehabilitation hospitals average 10.44 times, which have slower bed turnover in these specialty hospitals. Regarding the average bed working days: the average number of working days in oncology hospitals was 338.4, in psychiatric hospitals, was 323.9, and in chest hospitals was 320.1, and the average number of working days in these specialized hospitals was higher; the average number of working days in cosmetology hospitals was the lowest at 58.2 days. Regarding the bed utilization rate: tumor hospitals and psychiatric hospitals had higher bed utilization rates of 92.71% and 88.75%, respectively; while beauty hospitals and stomatology hospitals had lower bed utilization rates of 15.93% and 22.01%, respectively. The average length of stay in beauty hospitals, eye hospitals, and plastic surgery hospitals was shorter, at 2.6, 3.3, and 4.4 days, respectively; and the average length of stay in leprosy hospitals was the longest, at 63.6 days on average See Table 6; Fig. 6.

**Table 6:** Utilization of beds in various types of specialized hospitals nationwide, 2021

Mechanism classification	Number of bed turns (times)	Rank	Average hospital bed working days (days)	Rank	Utilization rate of hospital beds (%)	Rank	The average length of stay (day)	Rank	Rank Sum Ratio	Probit
Stomatological Hospital	11.48	4	80.3	2	22.01	2	6.7	14	0.2737	3.718
Eye Hospital	40.31	18	142.5	6	39.03	6	3.3	19	0.5759	5.000
Otolaryngology Hospital	32.23	15	178.5	8	48.90	8	5.3	17	0.5997	5.126
Tumor Hospital	43.08	19	338.4	20	92.71	20	7.8	13	0.9885	7.241
Cardiovascular Hospital	30.87	14	256.6	14	70.29	14	8.2	12	0.7477	5.674
Chest Hospital	33.91	17	320.1	18	87.69	18	9.6	10	0.8939	6.645
Hematology Hospital	32.33	16	314.1	17	86.06	17	9.6	9	0.8724	6.036
Obstetrics and Gynecology Hospital	28.07	12	160.9	7	44.08	7	5.5	16	0.5388	4.615
Children’s Hospital	43.23	20	282.7	15	77.45	15	6.5	15	0.8766	6.282
Psychiatric Hospital	5.29	2	323.9	19	88.75	19	55.3	2	0.6430	5.385
Infectious Disease Hospital	16.57	7	239.2	12	65.53	12	14.3	4	0.6166	5.253
Dermatology Hospital	14.06	5	130.6	5	35.78	5	8.6	11	0.3888	4.158
Tuberculosis Hospital	28.71	13	301.1	16	82.48	16	10.8	6	0.8220	5.842
Leprosy Hospital	0.88	1	92.4	3	25.30	3	63.6	1	0.1208	3.355
Occupational Disease Hospital	14.43	6	193.6	9	53.04	9	13.5	5	0.5114	4.476
Orthopaedic Hospital	22.52	10	234.4	11	64.22	11	10.1	8	0.6496	5.524
Rehabilitation Hospital	10.44	3	241.0	13	66.03	13	21.1	3	0.5710	4.874
Plastic Surgery hospital	25.49	11	112.9	4	30.94	4	4.4	18	0.4268	4.326
Beauty Hospital	19.32	9	58.2	1	15.93	1	2.6	20	0.2815	3.964
Other Specialized Hospitals	18.64	8	205.6	10	56.33	10	10.2	7	0.5674	4.747

The analysis found that RSR was significantly and linearly positively correlated with Y (*r* = 0.959, *P* = 0.000), and the linear regression equation was: RSR=-0.546 + 0.224Y. By categorizing the bed utilization efficiency of various specialty hospitals in each region in 2021, it was found that the rank-sum ratios of bed utilization efficiency of the oncology hospitals, the thoracic hospitals, the hematology hospitals, and the children’s hospitals were ≥ 0.797, which indicating a high utilization rate; infectious disease hospitals, dermatology hospitals, plastic surgery hospitals, rehabilitation hospitals, orthopedic hospitals, occupational disease hospitals, tuberculosis hospitals, other specialty hospitals, ophthalmology hospitals, psychiatric hospitals, obstetrics and gynecology (maternity) hospitals, cardiovascular hospitals, and otorhinolaryngology hospitals have rank sum ratios of bed utilization efficiencies ranging from 0.35 to 0.797, with average utilization efficiencies; and leprosy hospitals, cosmetology hospitals, Stomatology Hospital bed utilization efficiency rank sum ratio ≤ 0.3494, which is low utilization efficiency See Table 7.

**Table 7:** Breakdown of bed utilization efficiency by specialty hospitals nationwide, 2021

Category	Rank Sum Ratio(RSR)	Y(Probit)	Specific classification
Category I (high utilization efficiency)	≥ 0.797	≥ 6	Tumor Hospital, Chest Hospital, Hematology Hospital, Children’s Hospital
Category II (general utilization efficiency)	> 0.35 and < 0.797	> 4 and < 6	Infectious Disease Hospital, Dermatological Hospital, Plastic Surgery Hospital, Rehabilitation Hospital, Orthopedic Hospital, Occupational Disease Hospital, Tuberculosis Hospital, Other Specialized Hospitals, Ophthalmic Hospital, Psychiatric Hospital, Obstetrics and Gynecology Hospital, Cardiovascular Hospital, Otolaryngology Hospital
Category III (Low utilization efficiency)	≤ 0.3494	≤ 4	Leprosy Hospitals, Beauty Hospitals, dental hospitals

## Discussion

### Enhancing equity in health resource inputs in the East, middle, and West

In 2021, the National Health and Wellness Commission issued the Guiding Principles for Planning the Establishment of Medical Institutions (2021–2025), which made it clear that the establishment of medical institutions should be macro-regulated by the number of beds per 1,000 population and other major indicators, and that the target value of the number of beds per 1,000 population at the national level in 2025 would be 7.4 to 7.5 beds. From the results of the comparative analysis, the number of beds per 1,000 population in the central and western regions is close to the target value guided by the national plan, but the gap is larger in the eastern region. In terms of health technicians per 1,000 population, the eastern, central, and western regions are close. In terms of total health costs per capita and average total health costs per bed, the eastern region has higher inputs, while the central and western regions have lower inputs. From a national perspective, although the total investment in healthcare resources is lower overall, the distribution is more equitable, and there is still a gap in health cost investment between regions [6]. This is related to the scale of regional government financial expenditures: total health costs per capita and total health costs per bed in the eastern region are much higher than in the central and western regions. At the same time, there are also internal development imbalances within the regions.

At the national level, the basic balance of medical and healthcare staffing and beds shows that the country’s distribution of medical and healthcare resources is relatively equitable. China’s basic medical insurance policy is currently coordinated at the local and municipal levels, and the input of total health costs is mainly related to the level of the local economy and its capacity for development, so there is also an imbalance in the input of health costs. To solve these problems, government departments must strengthen the government’s responsibility, further enhance the coordination and allocation of medical resources, accelerate the expansion of high-quality medical resources and the balanced layout of the region; optimize and adjust the structure of the expenditure of health funds, and, while increasing the government’s financial investment, actively guide the participation of social capital to improve the quality and efficiency of medical care as a guide and expand the supply of medical service resources, with public medical institutions as the mainstay and non-public medical institutions as supplements. supplemented by public medical institutions, and expanding the supply of medical service resources.

### Improving the utilization rate of beds in primary healthcare institutions in various aspects

The purpose of evaluating the efficiency of hospital bed utilization is to improve the quality of diagnosis and treatment in hospitals, reduce hospitalization costs, reduce unreasonable costs, and maximize the allocation and utilization of health resources [7]. Dynamic analysis of the efficiency of hospital bed utilization, scientific optimization of the number of hospital beds, and the effective use of hospital bed resources is an effective way for medical institutions to improve service efficiency and service quality [8]. However, it is difficult to obtain a consensus on what the current standard hospital bed utilization rate should be maintained at. The U.S. National Health Plan guidelines for general hospitals, the minimum average bed occupancy rate of 80%, obstetrics for 75%, and pediatrics according to the size of the size from 65 to 75% varies. In the document on basic hospital standards issued by the General Office of China’s National Health Commission in 2019, the recommended appropriate range of bed utilization rate for tertiary hospitals is 85–93%, and the bed utilization rate for community hospitals should be ≥ 75%.

From the results of the data analysis, the bed utilization rate of national healthcare institutions is 69.82%, and the average bed utilization rate of hospitals in all regions of the country is 74.6%. The overall bed utilization rate is low. Among them, community health service centers (stations) are among the healthcare institutions with low bed utilization efficiency. This may be related to the following reasons: ① Compared with formal large hospitals such as general hospitals and Chinese medicine hospitals, the overall professionalism of health care personnel in community health service centers (stations) can not reach the level of patient demand; ② Objective reasons such as small sites and incomplete supporting equipment [9] make patients more willing to choose large hospitals to seek medical treatment; ③ The wage level of community health service centers (stations) is not high, and the space for promotion is limited, which makes them less attractive to patients. The lack of attraction for talent and serious staff turnover make the patients’ demand for medical treatment, not satisfied [10]; ④ The implementation of the two-way referral mechanism is still not perfect, and most of the primary healthcare institutions usually refer patients with acute, serious, and difficult diseases to higher-level general hospitals, but general hospitals seldom refer patients with common and frequent diseases to the primary healthcare institutions [11]; ⑤ China started to pilot the family doctor system in 2011, but the residents’ family doctor system has been implemented for a long time, and it is not easy for the patients to choose large hospitals. ⑤ China began to pilot the family doctor system in 2011, but the residents do not have a high evaluation of the system, and the phenomenon of signing but not contracting is common. In the administrative hierarchy system of allocating medical resources, the salary and employment prospects of general practitioners are difficult to make the doctors very enthusiastic [12].

Because of the above problems, it is recommended to improve the utilization rate of beds in primary healthcare institutions in many ways. First of all, it is necessary to improve the incentive mechanism of primary healthcare institutions, improve the management level, reduce personnel turnover, and at the same time open up the authority of physicians to work in multiple positions, flexibly utilize healthcare human resources, strengthen the geographical coordination of health care human resources, further enhance the feasibility of the implementation of the hierarchical diagnosis and treatment policy, and improve the policy of talent support. Secondly, it is necessary to accelerate the contracting system of general practitioners and implement the training and contracting process of family doctors, to lay a solid organizational foundation for the better development of primary healthcare institutions. Thirdly, information technology and Internet system should be utilized to improve the degree of informatization of primary healthcare institutions and provide technical guarantees for remote diagnosis and treatment by physicians. Fourthly, we should enrich the construction of the medical association system, promote the implementation of the hierarchical diagnosis and treatment system, accelerate the realization of the two-way referral system, and rationalize the allocation of medical resources.

### Adjusting the distribution of bed resources in specialized hospitals to achieve a balance between supply and demand

The analysis of research data found that the imbalance of bed utilization efficiency in specialized hospitals is reflected in the bed allocation and bed utilization rate of specialized hospitals, and the bed utilization efficiency of specialized hospitals such as leprosy hospitals, cosmetic hospitals, and stomatological hospitals belongs to the beds with low efficiency; whereas the bed utilization rate of oncology hospitals, thoracic hospitals, hematology hospitals, and children’s hospitals generally belongs to the high efficiency. The reason for the low efficiency of bed utilization in specialized hospitals such as leprosy hospitals may lie in the unsound referral system [13]; such hospitals usually receive patients with chronic diseases who are transferred away from general hospitals, and mainly provide long-term primary care and rehabilitation services; however, general hospitals usually lack the referral initiative for the downward sinking of patient resources [14]. At the same time, such hospitals also have problems such as outdated supporting diagnostic and treatment facilities, insufficient number of professional nursing physicians, inadequate hospital incentive mechanisms, and unstandardized service behaviors of healthcare personnel [15], which makes the efficiency of these specialized hospitals in receiving and treating patients somewhat limited [16]. The reason for the high bed utilization rate in oncology hospitals and thoracic hospitals lies in the fact that the diseases related to the patients seen in these hospitals are diseases with a long diagnostic and treatment course - types of diseases that are not easy to cure, and therefore the demand for beds is long, and the reason for the high bed utilization rate in children’s hospitals is the fact that children’s physical conditions are more fragile and prone to injuries, and they require more protection and rest.

When adjusting the number of beds in specialized hospitals, emphasis should be placed on increasing the number of beds in specialized hospitals with high bed utilization efficiency, such as oncology hospitals, thoracic hospitals, hematology hospitals, and children’s hospitals, etc. At the same time, emphasis should also be placed on supporting weak specialized hospitals, continuously introducing specialized medical and nursing personnel applicable to the characteristics of specialized hospitals, maximizing the satisfaction and efficiency of patients’ visits to specialized hospitals, and improving the service quality of specialized hospitals. The data show that there is a low number of beds in specialized hospitals. As for the health institutions with low bed utilization rates, as shown in the data, some of the beds can be transformed into beds suitable for hospitals to cooperate with doctors to complete the treatment of patients, thus enhancing the bed utilization rate of specialized hospitals and improving the service mode of specialized hospitals.

## Conclusion

In recent years, China has been increasing its investment in the development of health care year by year, whether in terms of the training of health professionals, the increase in the number of beds in health care institutions, or the financial investment in health care. However, the input of health expenses is not balanced. The utilization efficiency of beds in health institutions is on the low side, and medical efficiency in the eastern region is higher than in the central and western regions. To narrow the gap between the total health cost investment in the central and western regions and the eastern regions, and to improve the utilization efficiency of beds in health institutions, it is necessary to deepen the reform of China’s health care system from the perspective of enhancing the sense of responsibility of the government, increasing the government’s financial investment, balancing and coordinating resources, as well as strengthening the cultivation of human resources, strengthening the construction of information technology, etc.; and at the same time in the utilization efficiency of beds in different types of health institutions, it is necessary to further optimize the grassroots Meanwhile, in the bed utilization efficiency of different types of health institutions, it is also necessary to further optimize the bed utilization efficiency of primary health care institutions, improve the incentive mechanism of primary health care institutions, accelerate the cultivation and contracting process of general practitioners, and speed up the realization of the two-way referral system; the backwardness of hospital facilities, unsound mechanisms, and insufficient human resources in specialized hospitals, it is also necessary for the government to rationally arrange the amount of beds of various medical institutions, pay attention to the professional cultivation of medical and health care personnel, and promote a better health resource allocation and further enhancement of medical efficiency. It is also necessary for the Government to rationalize the number of beds in each medical institution and to focus on the specialized training of medical and health personnel, to promote better allocation of health resources and further enhance medical efficiency.

## Data Availability

The data comes from open database data.
